# Transglutaminase 2 and Ferroptosis: a new liaison?

**DOI:** 10.1038/s41420-023-01394-1

**Published:** 2023-03-09

**Authors:** Mara Gagliardi, Valentina Saverio, Federica Rossin, Manuela D’Eletto, Marco Corazzari

**Affiliations:** 1 Department of Health Sciences and Center for Translational Research on Autoimmune and Allergic Disease (CAAD), University of Piemonte Orientale, Novara, IT Italy; 2 Department of Biology, University of Rome Tor Vergata, Rome, IT Italy; 3 Interdisciplinary Research Center of Autoimmune Diseases (IRCAD), University of Piemonte Orientale, Novara, IT Italy

**Keywords:** Cell death, Cell signalling

Dear Editor,

The recently discovered form of non-apoptotic cell death program named Ferroptosis [1] is receiving more and more attention, as stated by increasing number of publications, due to its involvement in both physiological and pathological processes [2].

As new discovered process, the molecular mechanisms characterizing this new form of cell death are still largely unknown, thus fascinating an increasing number of scientists. The main signaling pathway is characterized by iron-dependent intracellular generation and accumulation of lipid peroxides (Lipid-ROS), which represent the main executioners of the process [1]. However, cells evolved certain pro-survival pathways to escape this cell death process, through Lipid-ROS detoxification, such as: (i) Gpx4 activity, the only known glutathione peroxidase able to reduce Lipid-ROS, using GSH as a co-factor [3]; (ii) the FSP1 (also known as AIFM2) pathway, characterized by lipid peroxides reduction through Coenzyme Q10 oxidation, which will be reduced, in turn, by FSP1 and NAD(P)H [4]; (iii) AKRs, which reduces Lipid-ROS into lower reactive molecules [5, 6]; and (iv) the cofactor GCH1/BH4 system, a potent endogenous radical-trapping antioxidant pathway [7].

Very recently, the autophagic process has also been linked to ferroptosis, since selective degradation of ferritin (through a process known as ferritinophagy), mediated by the carrier NCOA4, increases the intracellular labile iron pool (LIP), thus contributing to Lipid-ROS generation, and cell death [8].

However, although lipid peroxides represent the main executioners of this form of cell death, the molecular mechanism is still largely unknown, together with the role of other factors involved in the overall signaling pathway governing the regulation of ferroptosis.

In this respect, we found the potential role of TG2 (Transglutaminase 2) in the modulation of this form of cell death. Indeed, we found that TG2 ko MEFs (Suppl. S1) are resistant to ferroptosis induced by both RSL3 (a Gpx4 inhibitor) and Erastin (a System X_C_- inhibitor), compared to parental wt cells (Fig. 1A and Suppl. S2), although no difference in basal expression of SLC7A11 (target of ERA; Suppl. S3A) was observed, while decreased basal expression of GPX4 (target of RSL3; Suppl. S3B) was observed in TG2 ko cells, which should increase the sensitivity of cells to ferroptosis. Interestingly, although the accumulation of Lipid-ROS was promptly observed in wt MEFs exposed to RSL3, no lipid peroxidation was observed in TG2 ko counterpart (Fig. 1B), thus indicating that a process conferring resistance to ferroptosis is at work. Moreover, the analysis of basal expression of the downstream pro-ferroptotic marker ACSL4, responsible for PUFA introduction into cell membranes as PL-PUFA (combined with LPCAT3 activity), was slightly increased in TG2 ko cells compared to parental wt MEFs (Suppl. S4).

**Fig. 1:**
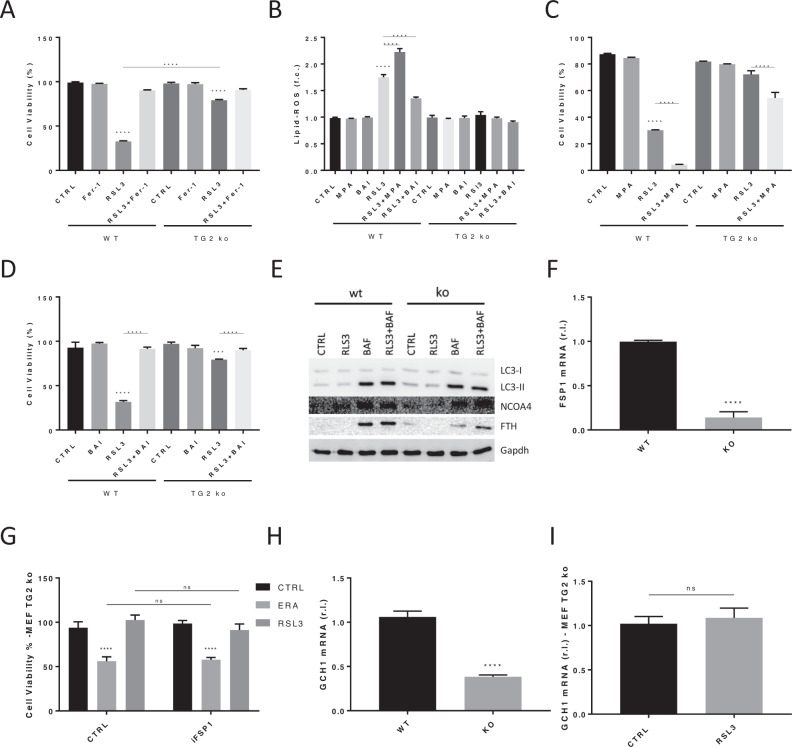
TG2 ko MEFs are resistant to Ferroptosis. **A** MEF wt and TG2 ko were exposed to 20 nM RSL3 alone or in combination with Fer-1 (10 μM) and cell viability was evaluated after 18 h, in cells stained with FDI/PI, by flow cytometry. **B** MEF wt and TG2 ko were treated as indicated and Lipid-ROS were evaluated after 8 h, by cytofluorimetric analysis of BODIPY C11 stained cells. **C** & **D** Cells were treated as indicated and cell viability was evaluated as in A. **E** MEF wt and TG2 ko were cultivated in complete medium ± BAF (5 nM) or exposed to RSL3 (20 nM) ± BAF and Autophagy was evaluated as LC3 conversion, while Ferritinophagy was evaluated by measuring the protein levels of FTH and NCOA4. **F** Basal expression of FSP1 was evaluated in wt and TG2 ko MEFs by qPCR. **G** Cell viability was evaluated as in B in TG2 ko MEF cells exposed 18 h to RSL3 (20 nM) alone or in combination with iFSP1 (6 μM). **H** Basal expression of GCH1 was evaluated in both wt and TG2 ko MEFs by qPCR. **I** TG2 ko MEFs were treated or untreated with 20 nM RSL3 and GCH1 expression was evaluated after 8 h, by qPCR. Data are representative of three independent experiments performed in triplicate. Histograms represent mean ± s.d.; *p < 0.05; **p < 0.01; ***p < 0.001; ****p < 0.0001; ns not statistically significant. Gapdh was used as loading control in IB reported in E.

We recently demonstrated the role of members of the AKRs enzymes superfamily (AKR1C1-3) in the resistance of metastatic melanoma cells to ferroptosis execution, through Lipid-ROS reduction [5, 6]. Importantly, the protective role of AKRs can efficiently be abrogated using the pan-inhibitor medroxyprogesterone (MPA), thus re-sensitizing cells to ferroptosis. Although mouse homologues of human AKR1C1-3 are still elusive, the use of MPA enhanced lipid peroxides (Fig. 1B) and toxicity (Fig. 1C and Suppl. S2) in wt MEF, compared to RSL3 alone. This possibly indicates a potential protective role of AKRs in wt MEFs response to pro-ferroptotic stimuli, at least in part. Conversely, MPA had no significant effect on both Lipid-ROS generation (Fig. 1B) and cell viability (Fig. 1D and Suppl. S2) of TG2 ko MEFs, indicating that AKRs are potentially not responsible for the resistance of these cells to ferroptosis. Moreover, we also identify ALOX15/12 as main factors responsible for Lipid-ROS generation in human metastatic melanoma cells [5]. By using the inhibitor Baicalein (BAI) in combination with RSL3 we completely prevented both Lipid-ROS generation and ferroptotic cell death also in MEF wt cells, while no effect was observed in TG2 ko cells (Fig. 1B, D). These data indicate the involvement of ALOX12/15 in the ferroptotic process induced by RSL3 also in MEF cells.

TG2 has been described actively involved in the autophagic process, possibly participating in the autophagosome-lysosome fusion [9]. Therefore, we evaluated whether the absence of TG2 might abrogate ferritinophagy in the RSL3-induced ferroptosis in MEF cells. To this aim, we evaluated both general autophagy and ferritinophagy in both wt and TG2 ko MEF cells, stimulated by nutrient deprivation (starvation) or RSL3 exposure. Our data clearly confirmed abrogated general autophagy in cells lacking TG2 (Suppl. S5), while no ferritinophagy was stimulated by RSL3 in wt or TG2 ko MEFs, as evidenced by no enhanced accumulation of both NCOA4 and FTH in cells exposed to RSL3 + BAF compared BAF alone (Fig. 1E). Of note, ferritinophagy induction results in increased intracellular labile iron pool (LIP) which, in turn, increases Lipid-ROS generation, through the Fenton reactions, and ferroptotic cell death [8]. On the contrary, increased ferritin expression would reduce LIP, thus inhibiting ferroptosis. Data reported in Fig. 1E show a slight enhanced FTH protein basal expression in TG2 ko cells compared to wt MEFs, possibly suggesting a TG2-dependent iron metabolism and/or LIP. Indeed, qPCR analysis confirmed a slight divergent iron metabolism in wt vs TG2 ko MEFs, as evidenced by increased expression of FTH (Suppl. S6A), decreased expression of FTL (Suppl. S6B), and a consistent decrease of DMT1 (Suppl. S6C) in cells lacking TG2. However, no differences in the intracellular Fe2+ content (LIP) was observed in cells lacking TG2 compared to wt MEFs.

Next, we explored the potential involvement of FSP1. To this aim, we evaluated the basal expression of this factor in wt and TG2 ko MEFs by both qPCR and western blotting analysis. Data reported in Fig. 1F clearly show lower expression of FSP1 in TG2 ko MEFs compared to parental cells at both mRNA (Fig. 1F) and protein (Suppl. S7) levels, thus indicating that the factor is not involved in the resistance of TG2 ko cells to ferroptosis.

Then, to confirm the above results, we exposed TG2 ko MEFs to RSL3 in combination with the FSP1 specific inhibitor iFSP1, and cell viability was evaluated. Data shown in Fig. 1G demonstrate no effect of iFSP1 on cell viability of MEFs treated with RSL3 or Erastin.

Finally, we also evaluated another antioxidant pathway with an anti-ferroptotic activity such as the GCH1/BH4 pathway. To this aim, we evaluated both basal and RSL3-stimulated expression of GCH1 in both wt and TG2 ko MEF cells. Results indicated lower GCH1 mRNA levels in MEF cells lacking TG2 (Fig. 1H), while no up-regulation was observed in TG2 ko MEFs exposed to RSL3 (Fig. 1I); the expression of GCLc was used as internal control (Suppl. S8).

Collectively, our results demonstrate that TG2 is involved in the signaling pathway responsible for ferroptosis execution, thus possibly representing a new actor in the ferroptotic cell death pathway, although its role is still elusive and thus requiring further studies.

## Supplementary information

Supplementary Figures

Supplementary Material

Original Data File

## Data Availability

All data are available in the main text or the supplementary materials.
