# Supplementary material for: Transglutaminase 2 and Ferroptosis: a new liaison?
Source: Cell Death Discov. 2023 Mar 9;9:88. doi: 10.1038/s41420-023-01394-1 (PMC9998634; doi:10.1038/s41420-023-01394-1)
Supplement: Supplementary file 1 — Supplementary Figures [file 41420_2023_1394_MOESM1_ESM.pdf]

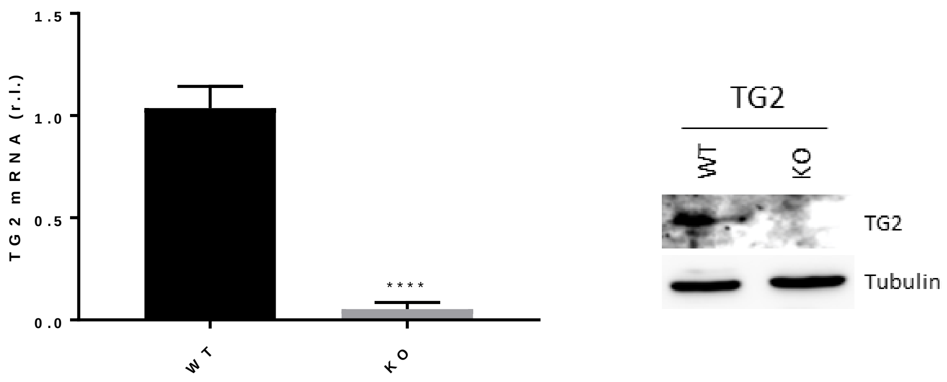

Supplementary S1. TG2 expression in wt and ko MEFs. The expression of TG2 was evaluated in wt and ko MEFs by both qPCR (left panel) and wb (right panel). Representative analysis of three independent expreriments. Histograms represent mean (triplicate) ± s.d.; **** p <0.0001; Tubulin was used as loading control (wb).

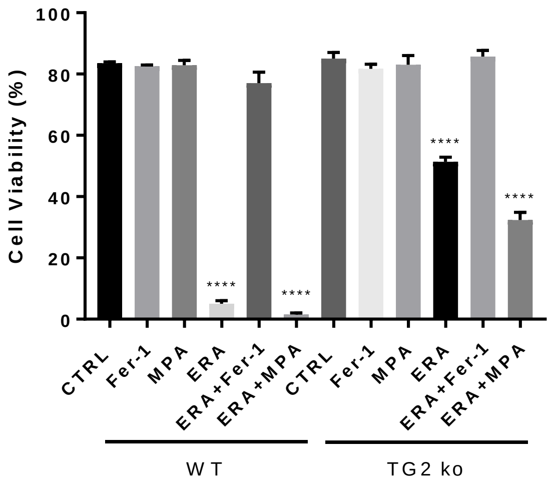

Supplementary S2. TG2 and sensitivity to Erastin. MEF wt and TG2 ko were exposed to Erastin (ERA; 0.5μM) alone or in combination with Ferrostatin-1 (Fer-1; 10 μM) or Medroxyprogesterone (MPA; 10 μM) and cell viability was evaluated after 24h, by FDA/PI stainng and fluorescence analysis by a Symphony flow cytometer (BD) plate-reader. Vehicle, Fer-1 or MPA were used as controls. Histograms represent mean ± s.d.; **** p <0.0001.

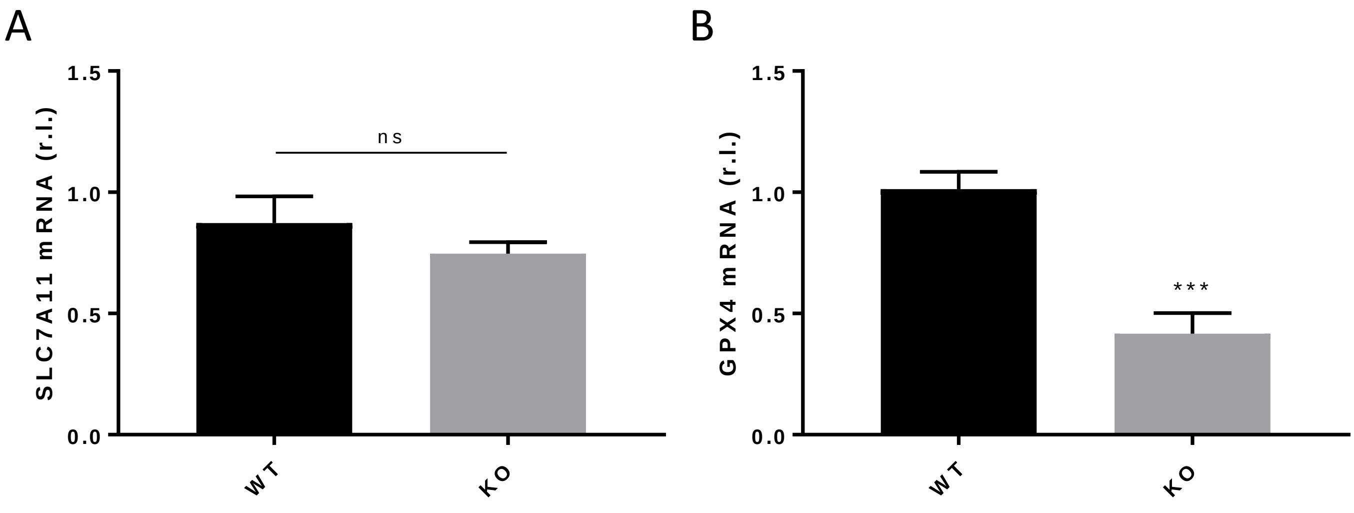

Supplementary S3. Basal expression of Ferroptosis biomarkers. The expression of SLC7A11 (A) and GPX4 (B) was evaluated in wt and ko MEFs by qPCR analysis. Histograms represent mean ± s.d.; *** p <0.001. The experiment was performed three times.

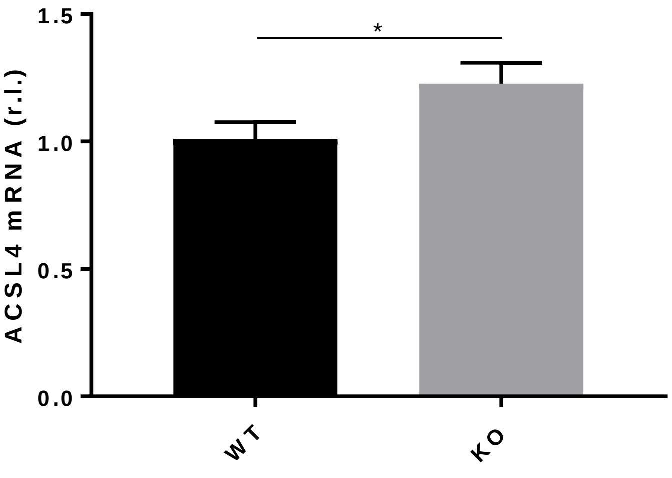

Supplementary S4. Basal expression of ACSL4. The expression of ACSL4 was evaluated in wt and ko MEFs by qPCR analysis. Histograms represent mean ± s.d.; *** p <0.05. The experiment was performed three times.

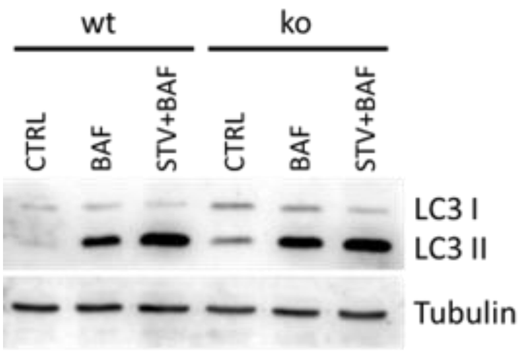

Supplementary S5. TG2 and autophagy. Wt and ko MEFs were cultivated 4h in complete medium in the absence (CTRL) or presence of Bafalomycin A1 (BAF) or in HBSS supplemented with Bafalomycin A1 (STV+BAF), and the conversion of LC3-I into LC3-II was evaluated by wb analysis. Tubulin was used as loading control. Images are representative of three independent experiments.

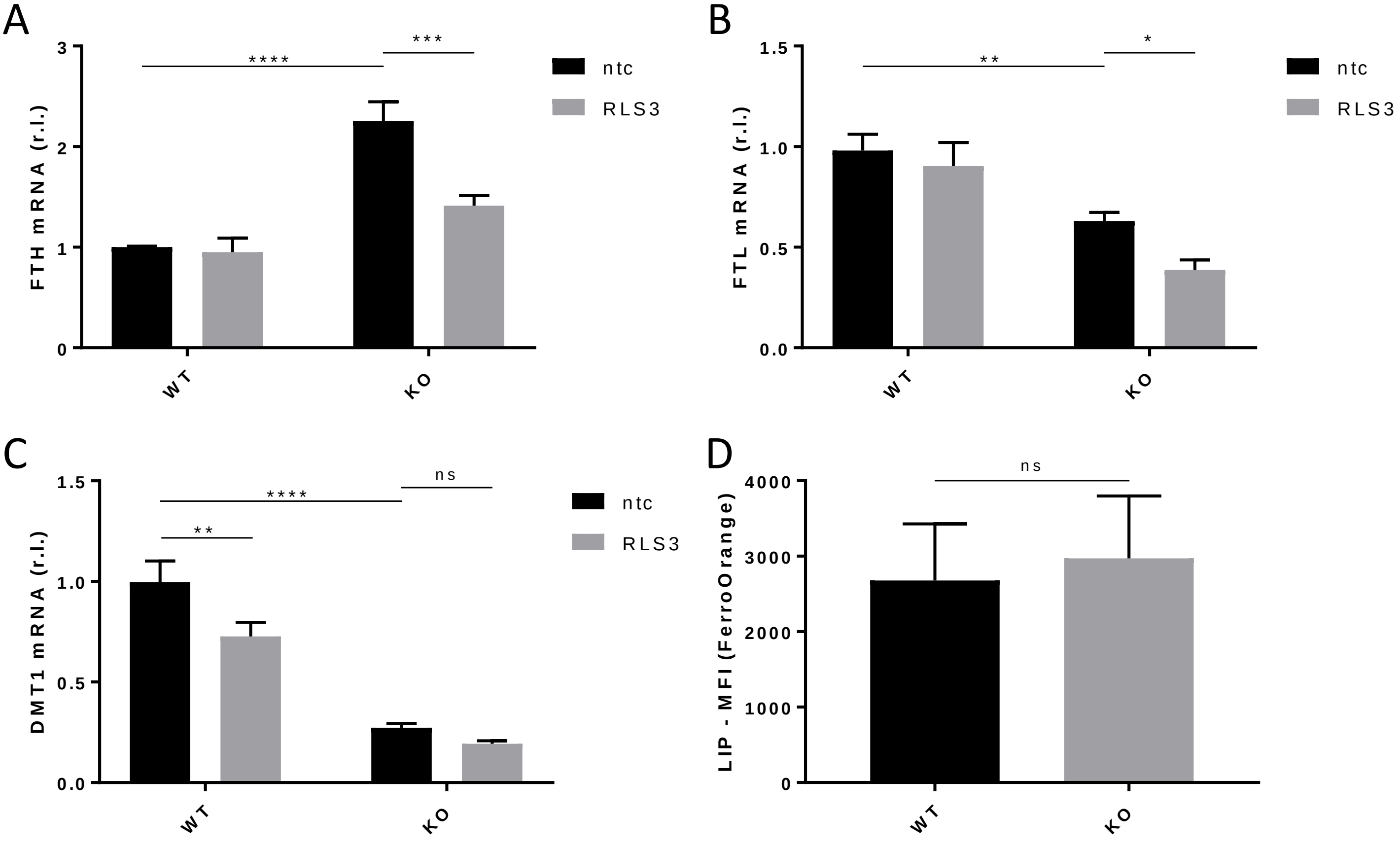

Supplementary S6. Iron metabolism. Iron metabolism was evaluated by measuring both basal and RSL3-stimulated expression of ferritin heavy chain (FTH; A), ferritin light chain (FTL; B), or DMT1 (C). While intracellular Fe^{2+} (labile iron pool – LIP) was detected with the FerroOrange probe (D). Histograms represent mean (triplicate) ± s.d. of experiments repeated three times; **** $p < 0.0001$; *** $p < 0.001$; ** $p < 0.01$; * $p < 0.05$; ns = not significant.

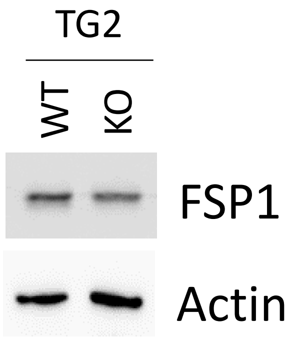

Supplementary S7. FSP1 expression in MEF cells. The expression of FSP1 was evaluated in wt and ko MEFs by wb analysis. Actin was used as loading control. Images are representative analysis of three independent expreriments.

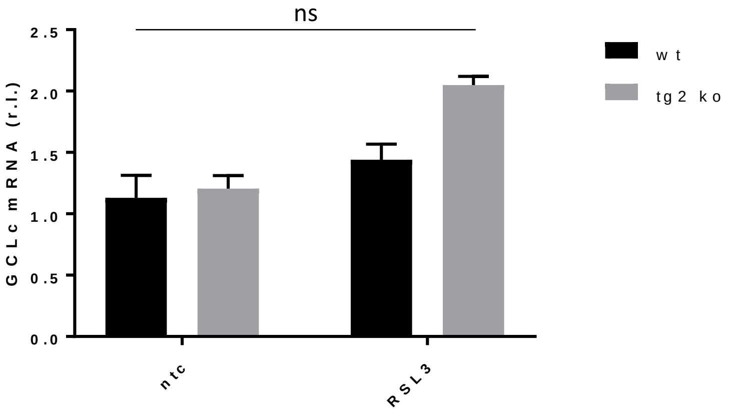

Supplementary S8. GCLc expression in MEF cells. The expression of GCLc was evaluated in wt and ko MEFs untreated (ntc) or treated 8h with 20nM RSL3, by qPCR. Histograms represent mean (triplicate) ± s.d. of experiments repeated three times; ns = not significant.
