# Supplementary material for: Transglutaminase 2 and Ferroptosis: a new liaison?
Source: Cell Death Discov. 2023 Mar 9;9:88. doi: 10.1038/s41420-023-01394-1 (PMC9998634; doi:10.1038/s41420-023-01394-1)
Supplement: Supplementary file 2 — Supplementary Material [file 41420_2023_1394_MOESM2_ESM.pdf]

Supplementary Material & Methods

Reagents

RSL3, Erastin, Ferrostatin-1, Baicalein, Medroxyprogesterone acetate, and Bafilomycin A1, Fluorescein diacetate, 7-AAD, protease inhibitors cocktail, non-fat dry milk, and Cell Lytic buffer were purchased from Merck; iFSP1 was from DBA; Dulbecco's Modified Eagle's Medium (DMEM), fetal bovine serum (FBS) and Penicillin/Streptomycin were from Euroclone; BODIPY C11 was from Invitrogen; TripleXtractor was from GRiSP; ExcelRT Reverse Transcriptase and Excel-Taq FAST qPCR SybrGreen were from SMOBIO; oligonucleotide based primers were from IDT.

Cells and treatments

WT and $TG2^{ko}$ MEF cells[1] were cultured in DMEM supplemented with 10% fetal bovine serum, 2mM L-glutamine, 1% penicillin/streptomycin solution at 37 °C under 5% CO_2.

Cells were treated with RSL3 0.02 μM, Erastin 0.5 μM, Medroxyprogesterone 10 μM, Baicalein 20 μM, Ferrostatin-1 10 μM, Bafilomycin A1 5 μM, iFSP1 6 μM.

Cell Viability assay

Fluorescein diacetate (FDA)/7AAD staining was used to identify and measure the percentage of live/dead cells. Briefly, cells were incubated (10 min) with PBS containing FDA (7 pg/ml) and 7AAD (50 ng/ml) and 10'000 events were acquired by flow cytometry. The percentage of FDA positive and 7AAD negative cells was measured and indicated as 'Cell Viability (%)'[2].

Lipid-ROS evaluation

Briefly, 1.5 × 105 cells were treatment as indicated and cells harvested at indicate time points. Then, cells were pelleted, washed by PBS, resuspended in BODIPY C11 (2 μM in PBS), incubated at 37 °C for 15 min in the dark, and 10.000 events were acquired by using a FACS Symphony cytometer (Becton-Dickinson). Data analysis was performed using the Flowing Software[3].

Western Blotting Analysis

Protein extraction was performed by using Cell Lytic buffer supplemented with a protease inhibitors cocktail plus phosphatases inhibitors (Na3VO4 1 mM; NaF 10 mM). Equal amount of protein lysates (20 μg) were subjected to SDS-Page separation and proteins electroblotted onto Nitrocellulose (Merck) membranes. 5% non-fat dry milk in PBS was used as blocking solutions, and indicated primary antibodies, in blocking solution, were incubated o.n. at 4°C. Appropriate HRP-conjugated secondary antibodies were diluted in blocking solution (1:5000) and incubate 1h at r.t. Westar ANTARES ECL kit (Cyanagen) was then used and signal acquired by a ChemiDocTMTouch (Bio-Rad), and analyzed by Image Lab software (Bio-Rad). Primary antibodies were: anti TG2 (1:500; Santa Cruz); anti-FSP1 (1:500; ProteinTech); anti-NCOA4 (1:500; Santa Cruz); anti-FTH (1:500; Santa Cruz); anti-LC3 (1:1000; Genetex); anti-Tubulin (1:5000; Santa Cruz); anti-Actin (1:2000; Merck); anti-Gapdh (1:5000; Santa Cruz). HRP-conjugated secondary antibodies were diluted in blocking solution (1:5000; Jackson ImmunoResearch)[4].

RT and Real-Time PCR (qPCR)

Total RNA was isolated by using TripleXtractor reagent and ExcelRT Reverse Transcrip-tase was used to produce cDNA, by using 2μg of total RNA. Quantitative PCR (qPCR) reactions were performed by using the Excel-Taq FAST qPCR SybrGreen and a CFX96 ther-mocycler (Bio-Rad). Primers sequences were designed by using the online IDT Pri-merQuest Tool software (IDT; https://eu.idtdna.com/Primerquest/Home/Index), and sequences reported below[5].

TG2	CCTGTTGCGTAAGGACATATT/GCCACTTCATCTTGCTCTAC
FSP1	CCTTCATGCTGGTGGATATG/GGTCGCAGAGTACGAATG
GCH1	CGAGATGGTGATTGTGAAGG/ACCAAGGACTTGCTTGTTAG
GCLc	TCGACCTGACCATCGATAA/GGGTGAGTGGGTCTCTAATA
SLC7A11	CTTTGTTGCCCTCTCCTG/CAGAGGAGTGTGCTTGTG
ACSL4	TAAGCCCAXTTCAGACAAAC/GGCTACAGCATGGTCAAATA
GPX4	TGGTCTGCCTGGATAAGT/TCGACTAGCTGAGTGTAGTT
FTH	CTACTGGAACTGCACAAACT/GGCTTTCACCTGCTCATT
FTL	GAAGCCAGCTGAAGATGA/CAGGGCATGAAGATCCAAA
DMT1	GCTGTCTTCCAAGATGTAGAG/GGATGGGTATGAGAGCAAAG
L34	GGTTGGGAAAGCACCTAAA/GACGTGCTTCTGTGTCTTAG

L34 mRNA level was used as an internal control, and comparative Ct method (ΔΔCt) was used for relative quantification of gene expression[6].

Measurement of Intracellular Fe^{2+} (LIP)

A FerroOrange fluorescent probe (SCT210, Merck) was used for the detection of intracellular Fe^{2+}. The cells were rinsed and incubated with 1 µM FerroOrange (in PBS) for 15min. Staining solution was discarded and cells were washed twice with PBS. Then, fluorescence was evaluated by flow cytometry (BD FACSymphony™ analyzer). The experiment was replicated three times in the study.

Statistical analysis

Experiments were performed in triplicate and repeated at least three times, and statistical analysis was performed using GraphPad software (GraphPad Software; GraphPad Prism 6). Student's t test or ANOVA was used to determine statistical significance.
A p-value of equal to or less than 0.05 was considered significant. mRNA expression levels were represented as 'fold change over control', r.l. relative levels. Histograms represent mean ± SD; **** $p < 0.0001$; *** $p < 0.001$; ** $p < 0.01$; * $p < 0.05$; ns non-significant.

References

1. Rossin F, Costa R, Bordi M, D'Eletto M, Occhigrossi L, Farrace MG, Barlev N, Ciccosanti F, Muccioli S, Chieregato L, Szabo I, Fimia GM, Piacentini M, Leanza L. Transglutaminase Type 2 regulates the Wnt/β-catenin pathway in vertebrates. Cell Death Dis. 2021 Mar 5;12(3):249
2. Monzani R, Gagliardi M, Clemente N, Saverio V, Pańczyszyn E, Santoro C, Yissachar N, Visciglia A, Pane M, Amoruso A, Corazzari M. The Gut-Ex-Vivo System (GEVS) Is a Dynamic and Versatile Tool for the Study of DNBS-Induced IBD in BALB/C and C57BL/6 Mice, Highlighting the Protective Role of Probiotics. Biology (Basel). 2022 Oct 27;11(11):1574
3. Gagliardi M, Cotella D, Santoro C, Corà D, Barlev NA, Piacentini M, Corazzari M. Aldo-keto reductases protect metastatic melanoma from ER stress-independent ferroptosis. Cell Death Dis. 2019 Nov 28;10(12):902
4. Pagliarini V, Giglio P, Bernardoni P, De Zio D, Fimia GM, Piacentini M, Corazzari M. Downregulation of E2F1 during ER stress is required to induce apoptosis. J Cell Sci. 2015 Mar 15;128(6):1166-79
5. Gagliardi M, Monzani R, Clemente N, Fusaro L, Saverio V, Grieco G, Pańczyszyn E, Yissachar N, Boccafoschi F, Corazzari M. A Gut-Ex-Vivo System to Study Gut Inflammation Associated to Inflammatory Bowel Disease (IBD). Biology (Basel). 2021 Jun 30;10(7):605
6. Ferrari E, Monzani R, Saverio V, Gagliardi M, Pańczyszyn E, Raia V, Villella VR, Bona G, Pane M, Amoruso A, Corazzari M. Probiotics Supplements Reduce ER Stress and Gut Inflammation Associated with Gliadin Intake in a Mouse Model of Gluten Sensitivity. Nutrients. 2021 Apr 7;13(4):1221
