# Supplementary material for: Transglutaminase 2 and Ferroptosis: a new liaison?
Source: Cell Death Discov. 2023 Mar 9;9:88. doi: 10.1038/s41420-023-01394-1 (PMC9998634; doi:10.1038/s41420-023-01394-1)

Western Blotting Images

WB LC3 – Fig.1E

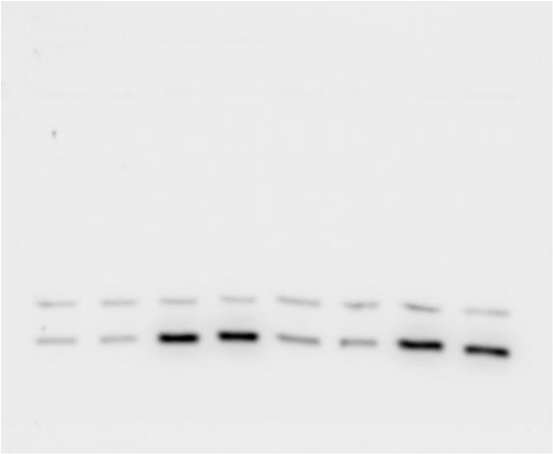

WB NCOA4 – Fig.1E

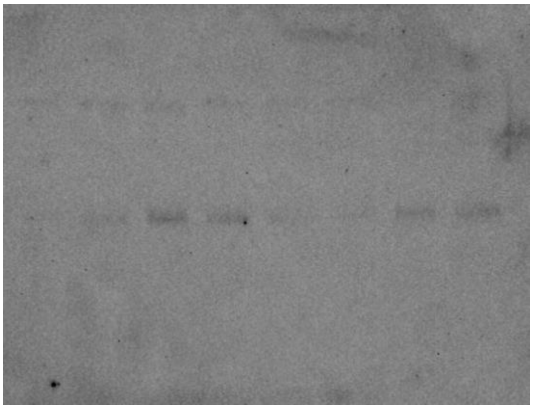

WB FTH – Fig.1E

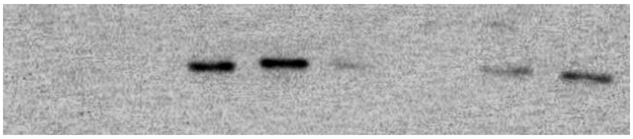

WB Gapdh – Fig.1E

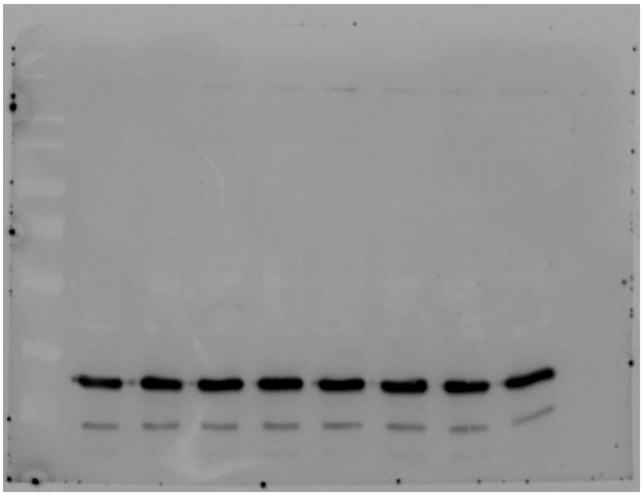

WB LC3 – S3

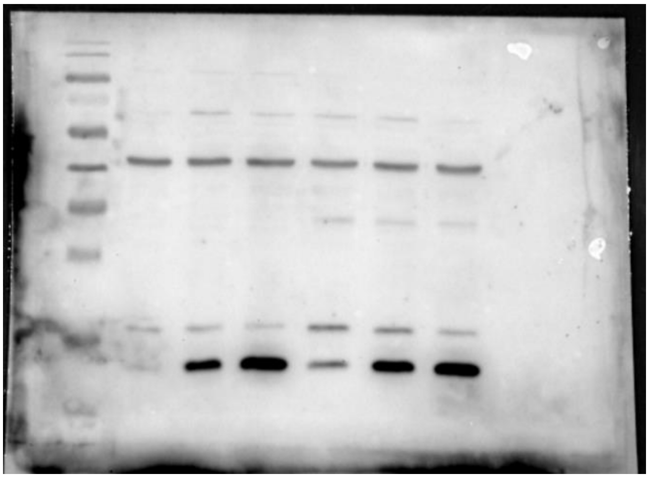

WB Tubulin – S3

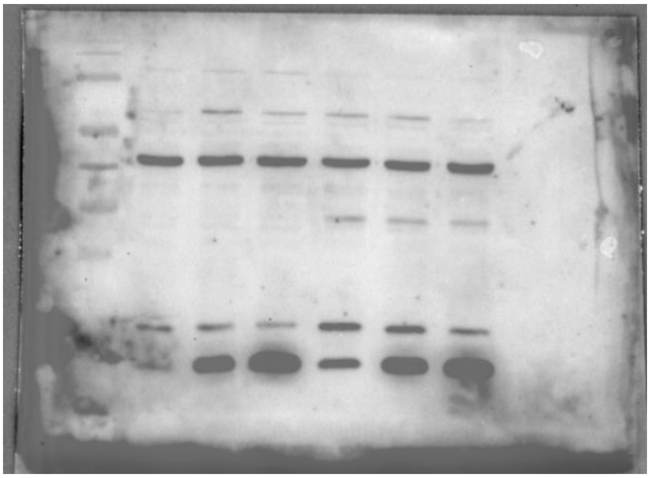

WB FSP1 – S4

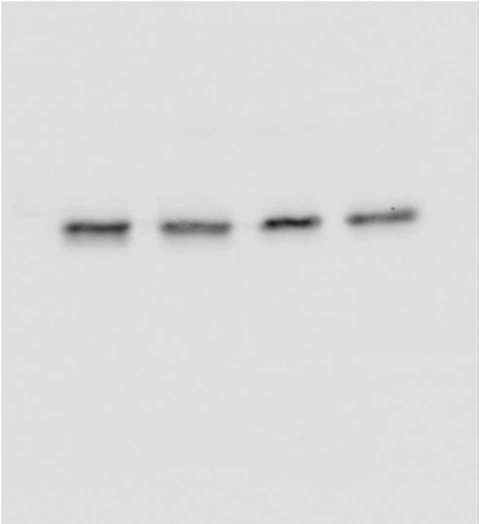

Supplement: Supplementary file 3 — Original Data File [file 41420_2023_1394_MOESM3_ESM.pdf]
